# *In Vitro* Study of Ineffective Erythropoiesis in Thalassemia: Diverse Intrinsic Pathophysiological Features of Erythroid Cells Derived from Various Thalassemia Syndromes

**DOI:** 10.3390/jcm11185356

**Published:** 2022-09-13

**Authors:** Woratree Kaewsakulthong, Thunwarat Suriyun, Sukanya Chumchuen, Usanarat Anurathapan, Suradej Hongeng, Suthat Fucharoen, Orapan Sripichai

**Affiliations:** 1 Department of Biochemistry, Faculty of Medicine Siriraj Hospital, Mahidol University, Bangkok 10700, Thailand; 2 Faculty of Medicine Ramathibodi Hospital, Mahidol University, Bangkok 10400, Thailand; 3 Thalassemia Research Center, Institute of Molecular Biosciences, Mahidol University, Nakhonpathom 73710, Thailand; 4 National Institute of Health, Department of Medical Sciences, Ministry of Public Health, Nonthaburi 11000, Thailand

**Keywords:** thalassemia, hemoglobin, globin, erythropoiesis, apoptosis, cell death

## Abstract

Defective hemoglobin production and ineffective erythropoiesis contribute to the pathophysiology of thalassemia syndromes. Previous studies in the field of erythropoiesis mainly focused on the severe forms of thalassemia, such as β-thalassemia major, while mechanisms underlying the pathogenesis of other thalassemia syndromes remain largely unexplored. The current study aimed to investigate the intrinsic pathophysiological properties of erythroid cells derived from the most common forms of thalassemia diseases, including α-thalassemia (hemoglobin H and hemoglobin H-Constant Spring diseases) and β-thalassemia (homozygous β^0^-thalassemia and β^0^-thalassemia/hemoglobin E diseases), under an identical *in vitro* erythroid culture system. Cell proliferation capacity, differentiation velocity, cell death, as well as globin synthesis and the expression levels of erythropoiesis modifying factors were determined. Accelerated expansion was found in erythroblast cells derived from all types of thalassemia, with the highest degree in β^0^-thalassemia/hemoglobin E. Likewise, all types of thalassemia showed limited erythroid cell differentiation, but each of them manifested varying degrees of erythroid maturation arrest corresponding with the clinical severity. Robust induction of HSP70 transcripts, an erythroid maturation-related factor, was found in both α- and β-thalassemia erythroid cells. Increased cell death was distinctly present only in homozygous β^0^-thalassemia erythroblasts and associated with the up-regulation of pro-apoptotic (Caspase 9, BAD, and MTCH1) genes and down-regulation of the anti-apoptotic BCL-XL gene.

## 1. Introduction

Thalassemia is considered the most common form of distressed congenital blood disorder characterized by attenuated production of globin chains, the subunits of hemoglobin (Hb) in red blood cells. Impairment of Hb formation and anemia are the main manifestations of this disease. Thalassemia falls into two major groups based on the type of globin chain defect caused by globin gene mutations: α-thalassemia and β-thalassemia. In Southeast Asia, homozygous β-thalassemia, β-thalassemia/hemoglobin E (HbE, *HBB*: c.79G > A; p.Glu27Lys), hemoglobin H (HbH; compound heterozygosity for α^0^-thalassemia and α^+^-thalassemia), hemoglobin H-Constant Spring (HbH-CS; compound heterozygosity for α^0^-thalassemia), and HbCS (*HBA2*: c.427T > C; p.Ter143GlnextTer31) are among the most common thalassemia syndromes [1]. Imbalance in α- to non-α-globin chain synthesis results in the precipitation of an excess unaffected globin chain, leading to the pathology of erythroid cells in association with ineffective erythropoiesis and hemolysis [2]. Interestingly, both α-thalassemia and β-thalassemia demonstrate phenotypic heterogeneity. The various clinical phenotypes of β-thalassemia rely on the broad diversity of β-globin gene mutations and modifiers involved in the degree of globin-chain imbalance. The interaction between these different variants provides a broad spectrum of β-thalassemia disease severity, ranging from nearly asymptomatic cases to transfusion-dependent patients with several complications [3,4]. The HbH-CS patients develop a severe form of the disease with hepatosplenomegaly, growth retardation, or transfusion-dependent hemoglobinopathy due to low α-globin chain synthesis and the presence of abnormal HbCS, whereas individuals with the classical HbH disease are usually less anemic and asymptomatic in steady state [5].

Impaired erythroid cell proliferation, differentiation, and viability affect the ineffective erythropoiesis in thalassemia patients [6]. Evidence of a defective phenotype of thalassemic erythroid cells was reported, but contrasting results were obtained from different experiments. In one *in vitro* study, erythroblasts derived from the bone marrow of homozygous β-thalassemia patients displayed low proliferation and accelerated early erythroid differentiation but with arrested terminal maturation [7]. Over-expansion [8,9,10,11] and impaired terminal maturation [10,11] of β-thalassemia/HbE erythroid cells were also reported. In contrast to β-thalassemia diseases, the HbH-CS erythroblasts displayed low cell proliferation and normal differentiation [12]. Furthermore, evidence of increased cell death and apoptosis has been found in erythroid precursors derived from the bone marrow aspirate of homozygous β-thalassemia [7,13,14], β-thalassemia/HbE, HbH-CS, and HbH diseases [15]. *In vitro* HbH-CS erythroblast culture also exhibited elevated apoptosis [12]. Conversely, another *in vitro* study of homozygous β-thalassemia erythroid cell culture showed increased cell proliferation without increased apoptosis [16]. Other previous *in vitro* culture studies reported premature apoptosis of β-thalassemia/HbE erythroblasts [17,18]. However, this phenomenon was not observed in a recent report [10].

Herein, the intrinsic pathophysiological properties of erythroid cells derived from homozygous β^0^-thalassemia, β^0^-thalassemia/HbE, HbH, and HbH-CS patients were investigated under the identical *in vitro* erythroid culture system. Patterns of heterogeneity in cell proliferation capacity, differentiation velocity, cell death, and Hb production were observed and correlated with clinical severity. Moreover, the study revealed gene expression profiles of molecular molecules associated with erythropoiesis and apoptosis.

## 2. Materials and Methods

### 2.1. Participant Samples

The study protocol was approved by the institutional review board of Mahidol University, Thailand (EOC No. 2016/139.0811). Peripheral blood of healthy normal subjects who did not have any anemic condition and thalassemia mutation (*n* = 5) and patients with thalassemia disease, including homozygous β^0^-thalassemia (*n* = 4), β^0^-thalassemia/HbE (*n* = 5), HbH (*n* = 5), and HbH-CS (*n* = 5), was collected after written informed consent by the participants. Participants were recruited between March and October 2017. Patient characteristics are shown in Supplemental Table S1. The analyses of hematological parameters (Advia2120 hematology analyzer; Siemens Healthcare Diagnosis, IL, USA), Hb typing (high-performance liquid chromatography (HPLC); VARIANT II hemoglobin testing system; Bio-Rad Laboratories, Hercules, CA, USA), and genotyping of α- and β-globin genes were performed as described previously [19]. Red blood cell parameters are shown in Table 1.

### 2.2. Hematopoietic CD34^+^ Cell Isolation and In Vitro Erythroid Cell Culture

CD34^+^ hematopoietic progenitor cells were isolated from peripheral blood by the magnetic bead selection method. Briefly, mononuclear cells were separated from blood by density gradient centrifugation principal (density = 1.077; LymphoprepTM, AXIS-SHIELD PoC AS, Oslo, Norway). CD34^+^ cells were selected using anti-CD34 magnetic microbeads positive selection kit (Miltenyi Biotech, Auburn, CA, USA) according to the manufacturer’s protocol. A two-phase culture system was adopted to drive CD34^+^ cells into erythroid lineage at 37 °C in 5% CO_2_ for 14 days as previously described [10]. Briefly, the basal medium was composed of Iscove’s Modified Dulbecco’s Medium (IMDM; Gibco, Grand Island, NY, USA) supplemented with 20% fetal bovine serum (FBS; Sigma-Aldrich, St Louis, MO, USA), 100 U/mL penicillin-streptomycin (Gibco), and 0.3 mg/mL human holotransferrin (PromoCell, Heidelberg, Germany). In phase I, cells were cultured in the basal media supplemented with 10 ng/mL interleukin 3 (IL-3; Cell Signaling Technologies, Beverly, MA, USA), 50 ng/mL stem cell factor (SCF; Cell Signaling Technologies), and 2 U/mL erythropoietin (EPO; CILAG GmbH, Zug, Switzerland) for 4 days. Later, the cells were cultured in phase II differentiation media, basal media containing 5 U/mL EPO, for 10 days. The cell count was performed on each day of culture by trypan blue exclusion assay utilizing hemocytometer under the light microscope. Cell morphology was observed by cytocentrifugation and modified Giemsa staining (Sigma, St. Louis, MO, USA), and pictures were captured under the Olympus CX31 light microscope (Olympus Corporation, Tokyo, Japan) equipped with a Canon D300 camera.

### 2.3. Flow Cytometric Analysis

Cell proliferation was investigated by staining the cells day 6 of culture with an intracellular fluorescent dye (CytoPainter Cell Tracking Staining; Abcam, Cambridge, MA, USA) and daily tracking the fluorescence intensity according to the manufacturer’s instructions. Erythroid cell differentiation during *in vitro* culture was monitored by stained cells with phycoerythrin (PE) conjugated anti-human transferrin receptor (CD 71; BD Bioscience, Pharmingen, San Diego, CA, USA) and allophycocyanin (APC) conjugated anti-human glycophorin A (CD235a; BD Bioscience), and fluorescent intensity was detected by the flow cytometer. Cell apoptosis was measured by stained cells days 10, 12, and 14 of culture with fluorescein (FITC) conjugated annexin V and propidium iodide (AnnV and PI; BD Bioscience) according to the manufacturer’s protocol. All flow cytometric analyses were performed on the BD FACSCalibur flow cytometer, and data analysis was interpreted on CellQuestTM software (BD Bioscience).

### 2.4. RNA Isolation and Quantitative RT-PCR Analysis

Total RNA was isolated using Trizol reagent (Invitrogen, Carlsbad, CA, USA) and converted to be cDNA by RevertAid First Strand cDNA Synthesis Kit (Thermo Fisher Scientific, Waltham, MA, USA) according to the constructor’s protocols. Quantitative RT-PCR assay was carried out with gene-specific primers and a SYBR™ Select Master Mix for CFX (Applied Biosystems™, Foster City, CA, USA) utilizing CFX Connect™Real-Time (Bio-Rad Laboratories, Hercules, CA, USA). The fold change in mRNA level was calculated using 2^−ΔΔCt^, and all the values were normalized to the expression of the ribosomal protein S18 (RPS18). Absolute quantification of globin mRNAs was performed as described previously [20].

### 2.5. Hemoglobin Quantification

Approximately 2 × 10^6^ erythroid cells were harvested on day 14 of the culture. The Hb type and quantification were characterized by HPLC (VARIANT II hemoglobin testing system, Bio-Rad Laboratories, Hercules, CA, USA). The percentage contents of different types of Hb were obtained by calculating and comparing the area of each peak.

### 2.6. Statistical Analysis

The data were expressed as mean ± standard deviation (SD). Statistical comparisons were made using unpaired *t*-tests. All the statistical analyses were performed using the Prism 6.0 computer program (GraphPad, La Jolla, CA, USA), and *p*-value less than 0.05 was considered significant.

## 3. Results

**Figure 1:**
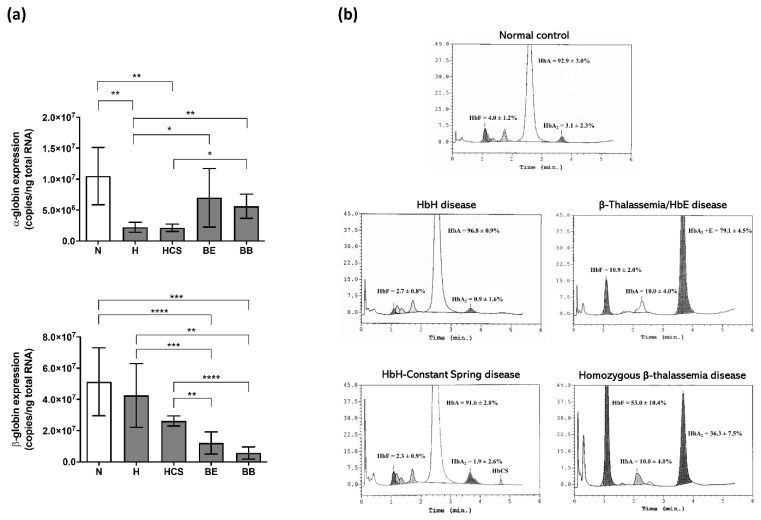
Defective globin production in thalassemic erythroid cells derived from *in vitro* culture. (**a**) Amount of α- and β-globin transcripts in cells day 10 of culture. (**b**) Chromatograms showing hemoglobin content in cells day 14 of culture. An error bar represents mean ± SD. * *p* ≤ 0.05, ** *p* ≤ 0.01, *** *p* ≤ 0.001, **** *p* ≤ 0.0001. N: normal, H: HbH disease, HCS: HbH-Constant Spring disease, BE: β^0^-thalassemia/HbE disease, BB: homozygous β^0^-thalassemia disease.

Defective globin production caused by the globin gene mutations was investigated by measuring the copy numbers of α- and β-globin mRNAs in the cells on day 10 of culture. As expected, α-globin mRNA was found significantly decreased in α-thalassemic erythroblasts (HbH and HbH-CS), and β-globin mRNA was also found significantly decreased in β-thalassemic erythroblasts (homozygous β^0^-thalassemia and β^0^-thalassemia/HbE) (Figure 1a). Of note, down-regulation of α-globin mRNA was observed in β-thalassemic erythroblasts, while slight down-regulation of β-globin mRNA was found in α-thalassemic erythroblasts. In addition, the amount and percentage of Hb content were determined at cell culture day 14 by HPLC (Figure 1b). HbA2 was slightly reduced in cells derived from HbH and HbH-CS patients. Importantly, HbCS was able to be detected by HPLC in this cell culture. Induction of fetal hemoglobin (HbF) was revealed in both cells derived from β^0^-thalassemia/HbE and homozygous β^0^-thalassemia patients.

**Figure 2:**
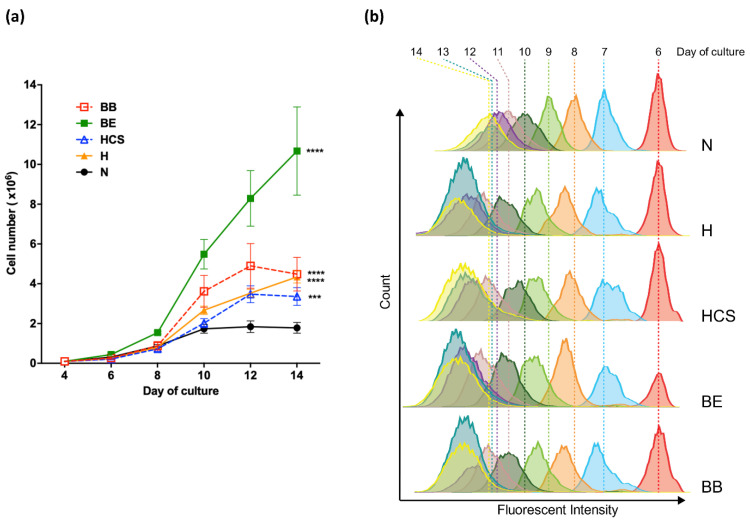
Elevated thalassemic erythroid cell expansion during *in vitro* erythropoiesis. (**a**) Cell number at the different time points of culture (day 4–14) of normal (N), HbH disease (H), HbH-CS disease (HCS), β^0^-thalassemia/HbE (BE), and homozygous β^0^-thalassemia (BB). *** *p* ≤ 0.001, **** *p* ≤ 0.0001 as compared to normal. (**b**) Dye dilution assay in erythroid cells day 6 of culture. The fluorescent intensity was captured by flow cytometer at day 6–14 of culture.

To determine cell expansion during *in vitro* erythropoiesis, cells were counted at the sequential time points from the same starting cells (1 × 10^5^ cells) at day 4 of culture. The expression of erythroid cell surface markers transferrin receptor (CD 71) and glycophorin A (CD235a), as well as cell morphology, were used to confirm erythroid lineage in the culture. Cell proliferation was not different in days 4 to 8 of culture, but a slightly higher cell number in β^0^-thalassemia/HbE erythroblasts was observed (Figure 2a). After day 8 of culture, all types of thalassemic erythroblasts started to show higher cell expansion over time when compared to normal controls. The erythroblasts derived from β-thalassemias revealed a higher cell proliferation rate than α-thalassemias; among these, the β^0^-thalassemia/HbE cell has the highest rate of cell proliferation. In addition, accelerated cell division was observed when monitored by dye dilution assay in all types of thalassemic erythroblasts, as shown by the lesser fluorescent intensities detected in comparison to normal cells on the same day of culture (Figure 2b). Of note, enhanced cell division began on day 8 of culture, consistent with the increased cell number determined by the cell counting method.

**Figure 3:**
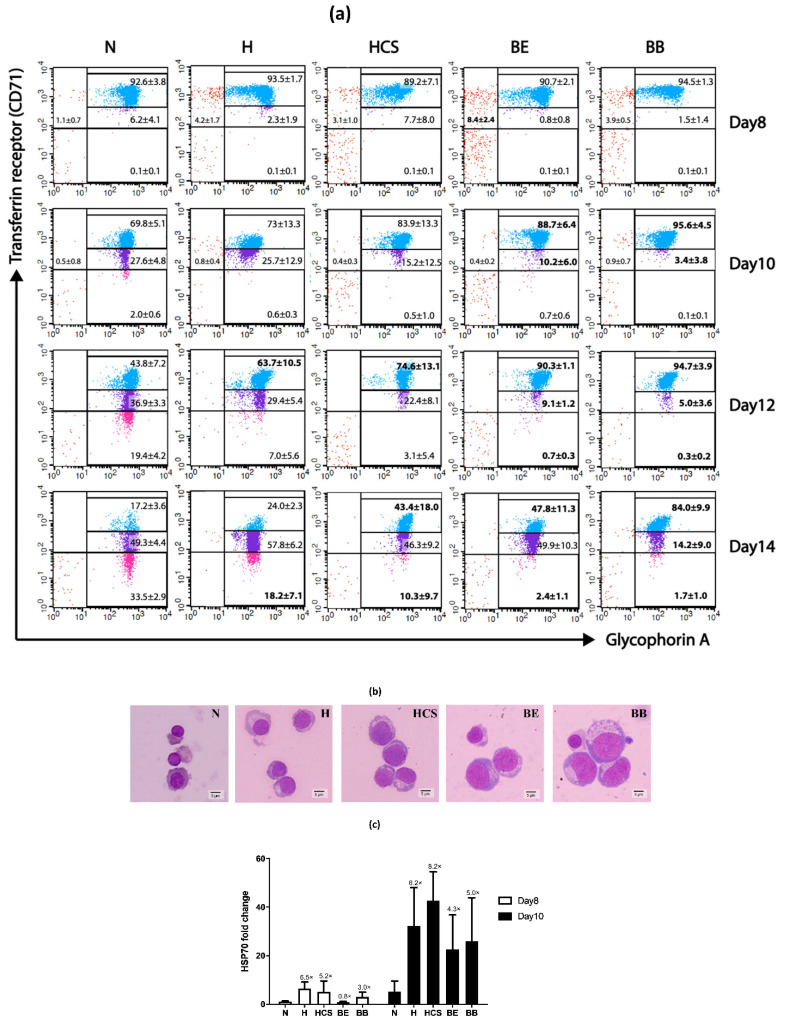
Delayed thalassemic erythroid cell differentiation during *in vitro* erythropoiesis. (**a**) Representative flow cytometry density dot plots of normal and thalassemic erythroid cells maturation during *in vitro* erythropoiesis. Red dots: CD71 high/GPA negative cells, blue dots: CD71 high/GPA positive cells, purple dots: CD71 moderate/GPA positive cells, pink dots: CD71 low/GPA positive cells. (**b**) Wright–Giemsa staining of cells harvested from day 14 culture. (**c**) Levels of HSP70 mRNA in erythroid cells days 8 and 10 of culture. Results from quantitative RT-PCR have been depicted from day 8 (white bar) and day 10 (black bar) of culture as relative fold change with the mean and SD value from independent subjects of each thalassemia group compared to the normal group. N: normal, H: HbH disease, HCS: HbH-Constant Spring disease, BE: β^0^-thalassemia/HbE disease, BB: homozygous β^0^-thalassemia disease.

In the present study, flow cytometry analysis of CD71 and CD235a expressions was conducted to monitor erythroid differentiation. CD235a was constantly expressed in erythroid lineage cells, whereas CD71 was expressed in erythroid committed progenitor cells and progressively declined during erythroid maturation. Impaired erythroid differentiation and terminal maturation arrest were observed in all types of thalassemia cells (Figure 3 and Table 2). Both HbH-CS and HbH erythroid cells displayed maturation arrest at days 12 and 14 of culture, but the greater delay was found in HbH-CS cells (CD71 low/GPA positive population on day 14 of culture = 10.3 ± 9.7% vs. 18.2 ± 7.1%, respectively; Table 2). An earlier delay in erythroid differentiation was observed in β-thalassemia cells (compared day 10 of culture with α-thalassemia and normal cells; Figure 3a). Extremely impaired cell differentiation was exhibited in the homozygous β^0^-thalassemia cells with approximately 84% of day 14 cells, which were the CD71 high/GPA positive population (Figure 3). Additionally, the expression levels of genes associated with erythroid maturation were investigated on cell culture day 10. The HSP70 mRNA was up-regulated in both α- and β-thalassemia erythroid cells (Figure 3c), while no significant differences in the levels of TGF-β receptor, ACTRIIA, ACTRIIB, GDF11, and FOXO3 transcripts were revealed between thalassemia and normal cells (Supplemental Figure S1).

**Figure 4:**
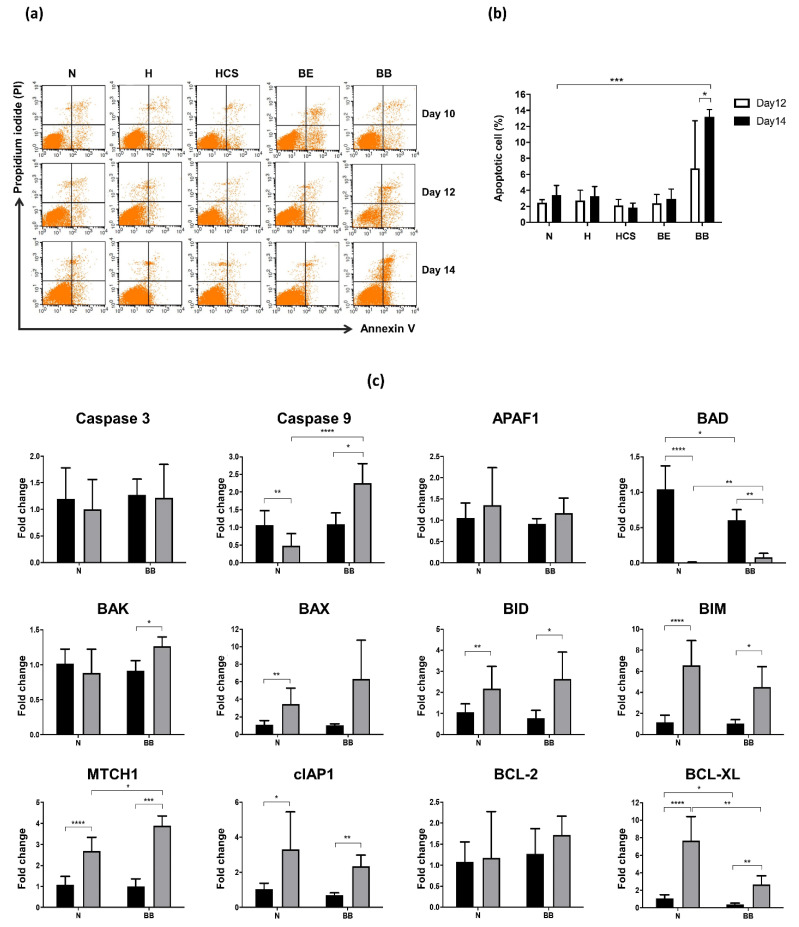
Increased apoptotic cells in the homozygous β^0^-thalassemia erythroid cells during *in vitro* culture. (**a**) Representative flow cytometry density dot plots of erythroblasts derived from normal control (N), HbH disease(H), HbH-CS disease (HCS), β^0^-thalassemia/HbE disease (BE), and homozygous β^0^-thalassemia disease (BB) on days 10, 12, and 14 of cell culture. (**b**) Percentage of erythroid cells undergoing apoptosis (Annexin V positive and PI positive population) at days 12 (white bar) and 14 (black bar) of culture. (**c**) Quantitative RT-PCR analysis of transcript levels of apoptotic related genes in days 10 (black bar) and 12 (gray bar) of culture. The results were calculated as a fold change to the mean of transcript levels of cells from normal control day 10 of culture. * *p* ≤ 0.05; ** *p* ≤ 0.01; *** *p* ≤ 0.001; **** *p* ≤ 0.0001.

Cell death and apoptosis were investigated on days 10, 12, and 14 of cell culture using Annexin V and PI labeling assay (Figure 4a). A significant increase in apoptotic cells (AnnV positive/PI positive population) was observed in homozygous β^0^-thalassemia cells on day 14 of culture (*p* < 0.0001; Figure 4b). However, apoptosis was not elevated in cells derived from other thalassemia syndromes. Furthermore, quantitative RT-PCR analysis of the known regulating factors involved in cell apoptosis was performed in mRNA extracts from days 10 and 12 of cell culture derived from homozygous β^0^-thalassemia patients and normal participants (Figure 4c). The candidate modifying molecules that included pro-apoptotic (Caspase 3, Caspase 9, APAF1, BAD, BAK, BAX, BID, BIM, and MTCH1) and anti-apoptotic (cIAP1, BCL-2, and BCL-XL) genes were investigated. During normal erythroid terminal differentiation, down-regulation of Caspase 9 and BAD expression and up-regulation of BAX, BID, BIM, MTCH1, cIAP1, and BCL-XL expression was revealed on days 12 and 14 of cell culture, while Caspase 3, APAF1, BAK, and BCL-2 transcripts remained unchanged during cell differentiation. A similar change in transcript levels was observed in homozygous β^0^-thalassemia cells, but marked up-regulation of Caspase 9 and BAK was found. Of note, the dramatic decrease in BAD transcript level on days 12 and 14 of culture was revealed in both normal and homozygous β^0^-thalassemia cells. Interestingly, significant down-regulation of anti-apoptotic BCL-XL gene but up-regulation of pro-apoptotic Caspase 9, BAD, and MITCH1 genes was observed on day 12 of homozygous β^0^-thalassemia cells compared to normal cells. No difference in transcript level was found in other genes of interest.

## 4. Discussion

This study revisits the correlation between the pathogenesis of thalassemia and the intrinsic erythroid phenotypes. Comparison of cell phenotypes from different thalassemia syndromes examined in the identical *in vitro* erythroid culture system suggests that the degree of defective erythropoiesis correlates with the severity of thalassemia syndromes. The homozygous β^0^-thalassemia erythroid cells displayed a significant failure to synthesize globin transcripts, with the highest degree of ineffective erythropoiesis resulting from accelerated proliferation accompanied by severely impaired terminal differentiation and marked apoptosis. Insufficient globin production and ineffective erythropoiesis were also observed in β^0^-thalassemia/HbE cells with a minor degree of defectiveness. Further, α-thalassemia erythroid cells displayed a remarkably lower degree of ineffective erythropoiesis compared to β-thalassemia, which correlated with the milder symptoms. Erythroid characteristics of HbH-CS were similar to HbH disease, but terminally mature HbH-CS cells experienced greater impairment. The membrane-bound oxidized α^CS^-globin chain leading to hemolysis contributes to a more severe phenotype in HbH-CS [21]. Although HbCS was detected in this *in vitro* cell culture system, this current study was unable to investigate a correlation between the existence of HbCS and impaired erythroid terminal maturation. It is known that hemolytic clinical phenotypes in thalassemia arise from an imbalance of globin chain synthesis. The level of α- to non-α-globin mRNAs in erythroblasts derived from β-thalassemia was higher than that of α-thalassemia, which is associated with more severe clinical symptoms in β-thalassemia. No significant difference in globin mRNA imbalance was found within each type of thalassemia syndrome. It is recommended that future studies should aim to examine imbalanced globin chain synthesis at the protein level among distinctive thalassemia syndromes.

Chiefly, the results revealed an accelerated expansion but limited differentiation in erythroblast cells derived from β-thalassemia patients, which is consistent with previous *in vitro* and *in vivo* studies carried out in β-thalassemic mice [22]. Notably high rates of proliferation of thalassemic erythroblasts were found in the early stage of erythroid cells, days 6–10 of culture, which expressed CD71 high/GPA negative and CD71 high/GPA positive corresponding to the BFU-E, CFU-E, proerythroblasts, and basophilic erythroblasts. Given that culture conditions were identical in all the experiments, the greater proliferative capacity found in thalassemic erythroblasts suggests a stronger response to external stimuli than normal cells could show. In response to chronic anemia, intrinsic mechanisms of thalassemic erythroblasts may be primed to undergo a higher expansion by increasing cell division rates and delaying differentiation of immature erythroblasts to compensate for the insufficient number of red blood cells. Unfortunately, this response might decrease the number of mature red blood cells. This study showed that an earlier and more delayed erythroblast differentiation, as well as a higher proliferation rate in β-thalassemic compared to α-thalassemic cells, was associated with higher anemic conditions in β-thalassemia. Recently, we reported alterations in several erythropoiesis modifying factors, such as GDF11, FOXO3, HSP70, and ACVR2A, with delayed terminal erythroid maturation in β^0^-thalassemia/HbE. The present study measured the expression levels of these factors in the earlier stage of cells and found a robust induction of the chaperone heat shock protein 70 (HSP70) in both β- and α-thalassemic cells. At different stages of erythropoiesis, HSP70 has distinct functions, ranging from modulating cell signaling to protein quality control activities. Thus, HSP70 dysfunction is associated with ineffective erythropoiesis that causes chronic anemia in several hematological disorders in humans [23]. Normal erythroid maturation requires a transient activation of caspase-3 and that HSP70 accumulates in the nucleus to protect the transcription factor GATA-1 from caspase-3 cleavage [24]. Inhibition of nuclear HSP70 accumulation by free α-globin chains leads to maturation arrest and apoptosis in β-thalassemia major erythroblasts [25]. Therapeutic options to ameliorate ineffective erythropoiesis involve restoring of terminal maturation through the HSP70 pathway. It was demonstrated that increasing the amount of nuclear HSP70 could rescue GATA-1 expression and improve terminal differentiation in β-thalassemia cells [25,26]. Our data showed a high level of HSP70 expression in all types of thalassemic cells, suggesting an intrinsic response to restore impaired erythroid differentiation. Interestingly, higher HSP70 expression was found in α-thalassemia erythroblasts compared to β-thalassemia. Nevertheless, the underlying mechanism for the induction of higher HSP70 and free β-globin chain expression in α-thalassemia erythroblasts has not been characterized. The protein expression of nuclear HSP70 protein was not investigated in this study.

Immature erythroid apoptosis is also the major contributor to ineffective erythropoiesis. This study demonstrated significantly increased levels of cell death and apoptosis in homozygous β^0^-thalassemia cells, but these were not found in cells derived from β^0^-thalassemia/HbE and α-thalassemia syndromes. Additionally, high erythroid cell death in homozygous β^0^-thalassemia is associated with decreased levels of anti-apoptotic BCL-XL (BCL2L1; BCL2 like 1) transcripts and increased levels of pro-apoptotic Caspase 9, BAD (BCL2 associated agonist of cell death), and MTCH1 (mitochondrial carrier 1) transcripts. The intrinsic apoptotic pathway acts through the mitochondria upon activation, and this signaling process is strictly regulated by the BCL-2 family proteins [27]. Cell survival under steady-state conditions is ensured by the anti-apoptotic proteins binding to the downstream pro-apoptotic effector proteins, BAX and BAK, thereby preventing them from activating the execution phase of apoptosis and inflicting mitochondrial damage [28]. BCL-XL can be found in the outer mitochondrial membrane and in soluble cytosolic homodimers. BCL-XL is up-regulated in human erythroblasts during terminal differentiation. Repression of BCL-XL severely impacted the survival of immature erythroid cells (CD71 high and GPA negative population) and their progression to late stages of maturation [29]. BCL-2 and BCL-XL are targets of the pro-apoptotic BAD, which specifically blocks the activity of both anti-apoptotic factors by forming heterodimeric complexes with either of the two proteins and displacing BAX [30,31]. BAD is a cell death regulator that constitutes a critical control point in the intrinsic pathway of apoptosis, which occurs through the dysregulation of the mitochondrial outer membrane permeabilization and the subsequent release of apoptogenic factors. The dramatically decreased expression levels of BAD during erythroid maturation were observed in both normal and thalassemia cells, suggesting its role in human erythropoiesis. MTCH1 is localized to the mitochondrion inner membrane and induces apoptosis independent of the pro-apoptotic proteins BAX and BAK [32]. Sequential activation of caspase enzymes plays a central role in the execution phase of cell apoptosis. Caspase 9 undergoes autoproteolytic processing and activation by the apoptosome, which is one of the earliest steps in the caspase activation cascade. Alterations of BCL-XL, BAD, MTCH1, and Caspase 9 expression were found in homozygous β^0^-thalassemic cells. However, further functional characterization of these factors is needed. Deeper insight into how they are regulated will help unveil the exact mechanisms that control and prevent cell death.

In conclusion, this *in vitro* erythroid culture study suggests that enhanced erythroblast proliferation in parallel with limited cell differentiation and maturation arrest contributes to ineffective erythropoiesis in both α- and β-thalassemia diseases. Various degrees of erythroid defects during erythropoiesis were observed in distinct thalassemia syndromes, corresponding to anemia level and clinical phenotypes. The findings reinforce the correction of ineffective erythropoiesis by inducing erythroid differentiation, which might emerge as a universal treatment for thalassemia syndromes. Additionally, balancing the pro- and anti-apoptotic factors presents an alternative approach to relieving ineffective erythropoiesis in homozygous β-thalassemia.

## Figures and Tables

**Table 1:** Red blood cell parameters in thalassemia patient and healthy normal participant.

Study Participants	No.	RBC (×10^6^ cells/uL)	Hb (g/dL)	Hct (%)	MCV (fL)	MCH (pg)	MCHC (g/dL)
Healthy normal	5	4.5 ± 0.2	12.8 ± 0.8	38.5 ± 1.8	86.5 ± 3.5	28.6 ± 1.3	33.1 ± 0.6
Hemoglobin H disease	5	5.6 ± 0.5	9.1 ± 1.2	31.5 ± 3.3	55.8 ± 2.7	16.3 ± 1.4	29.1 ± 2.1
Hemoglobin H-Constant Spring disease	5	4.0 ± 0.6	7.8 ± 0.5	30.9 ± 4.7	77.4 ± 4.3	19.8 ± 2.6	25.6 ± 2.7
β^0^-Thalassemia/hemoglobin E disease	5 *	3.9 ± 0.3	8.3 ± 1.1	26.9 ± 2.6	68.2 ± 4.1	21.2 ± 2.4	31.0 ± 2.1
Homozygous β^0^-thalassemia disease	4 *	2.7 ± 0.8	7.0 ± 2.0	21.0 ± 5.6	77.8 ± 3.2	26.0 ± 0.9	33.5 ± 1.9

RBC, red blood cell; Hb, hemoglobin; Hct, hematocrit; MCV, mean corpuscular volume; MCH, mean corpuscular hemoglobin; MCHC, mean corpuscular hemoglobin concentration. Data are presented as mean ± SD. * Transfusion-dependent thalassemia patients.

**Table 2:** Percentage of erythroid subsets of normal and different thalassemia types at different day of culture. * *p* ≤ 0.05 as compared to normal.

Day of Culture	Erythroid Populations	Normal	HbH Disease	HbH-CS Disease	β^0^-Thalassemia/ HbE Disease	Homozygous β^0^-Thalassemia
Day 8	CD71 high/GPA negative	1.1 ± 0.7	4.2 ± 1.7	3.1 ± 1.0	8.4 ± 2.4 *	3.9 ± 0.5
	CD71 high/GPA positive	92.6 ± 3.8	93.5 ± 1.7	89.2 ± 7.1	90.7 ± 2.1	94.5 ± 1.3
	CD71 moderate/GPA positive	6.2 ± 4.1	2.3 ± 1.9	7.7 ± 8.0	0.8 ± 0.8	1.5 ± 1.4
	CD71 low/GPA positive	0.1 ± 0.1	0.1 ± 0.1	0.1 ± 0.1	0.1 ± 0.1	0.1 ± 0.1
Day 10	CD71 high/GPA negative	0.5 ± 0.8	0.8 ± 0.4	0.4 ± 0.3	0.4 ± 0.2	0.9 ± 0.7
	CD71 high/GPA positive	69.8 ± 5.1	73.0 ± 13.3	83.9 ± 13.3	88.7 ± 6.4 *	95.6 ± 4.5 *
	CD71 moderate/GPA positive	27.6 ± 4.8	25.7 ± 12.9	15.2 ± 12.5	10.2 ± 6.0 *	3.4 ± 3.8 *
	CD71 low/GPA positive	2.0 ± 0.6	0.6 ± 0.3	0.5 ± 1.0	0.7 ± 0.6	0.0 ± 0.1
Day 12	CD71 high/GPA negative	0.0	0.0	0.0	0.0	0.0
	CD71 high/GPA positive	43.8 ± 7.2	63.7 ± 10.5 *	74.6 ± 13.1 *	90.3 ± 1.1 *	94.7 ± 3.9*
	CD71 moderate/GPA positive	36.9 ± 3.3	29.4 ± 5.4	22.4 ± 8.1	9.1 ± 1.2 *	5.0 ± 3.6 *
	CD71 low/GPA positive	19.4 ± 4.2	7.0 ± 5.6	3.1 ± 5.4	0.7 ± 0.3 *	0.3 ± 0.2 *
Day 14	CD71 high/GPA negative	0.0	0.0	0.0	0.0	0.0
	CD71 high/GPA positive	17.2 ± 3.6	24.0 ± 2.3	43.4 ± 18.0 *	47.8 ± 11.3 *	84.0 ± 9.9 *
	CD71 moderate/GPA positive	49.3 ± 4.4	57.8 ± 6.2	46.3 ± 9.2	49.9 ± 10.3	14.2 ± 9.0 *
	CD71 low/GPA positive	33.5 ± 2.9	18.2 ± 7.1 *	10.3 ± 9.7 *	2.4 ± 1.1 *	1.7 ± 1.0 *

## Data Availability

Not applicable.

## Supplementary Materials

The following supporting information can be downloaded at: https://www.mdpi.com/article/10.3390/jcm11185356/s1, Figure S1: Quantitative RT-PCR analysis of transcript levels of erythropoiesis modifying factors in day 8 (white bar) and day 10 (black bar) of culture as relative fold change with the mean and SD value from independent subjects of each thalassemia group compared to the normal group. N; normal, H; HbH disease, HCS; HbH-Constant Spring disease, BE; β^0^-thalassemia/HbE disease, BB; homozygous β^0^-thalassemia disease. Table S1: Thalassemia patients’ characteristics.
