# Supplementary material for: In Vitro Study of Ineffective Erythropoiesis in Thalassemia: Diverse Intrinsic Pathophysiological Features of Erythroid Cells Derived from Various Thalassemia Syndromes
Source: J Clin Med. 2022 Sep 13;11(18):5356. doi: 10.3390/jcm11185356 (PMC9504363; doi:10.3390/jcm11185356)

Supplemental Table S1. Thalassemia patients' characteristics.

	Age (yr)	Gender	Hb level (g/dl) *	Age at onset (yr)	Age at first transfusion (yr)	Requirement for transfusion #	Size of spleen (cm) or splenectomized	Growth development †	
								Height	Weight
**Hemoglobin H disease**									
H-1	10	Female	7.9	4	None	None	2	91%	78%
H-2	14	Female	8.4	5	9	Rare	< 1	97%	86%
H-3	9	Male	9.5	3	None	None	1	100%	100%
H-4	13	Male	9.7	6	None	None	6	93%	85%
H-5	14	Female	9.8	1	None	None	5	98%	87%
**Hemoglobin H-Constant Spring disease**									
HCS-1	9	Female	6.9	5	5	Frequent	8	97%	90%
HCS-2	8	Female	7.3	1	5	Frequent	Splenectomized	95%	95%
HCS-3	11	Male	7.9	6	6	Occasional	7	95%	84%
HCS-4	8	Male	8.2	6	7	Occasional	< 1	98%	94%
HCS-5	7	Male	8.5	4	5	Occasional	< 1	100%	100%
**β^0-Thalassemia/hemoglobin E disease**									
BE-1	10	Female	6.4	4	5	Frequent	7	89%	75%
BE-2	8	Female	6.8	1	5	Frequent	Splenectomized	95%	95%
BE-3	11	Female	6.9	1	1	Frequent	4	92%	72%
BE-4	15	Male	7.9	4	4	Frequent	Splenectomized	98%	73%
BE-5	12	Male	8.1	4	5	Frequent	5	94%	86%
**Homozygous β^0-thalassemia disease**									
BB-1	11	Female	4.2	1	1	Frequent	Splenectomized	86%	81%
BB-2	7	Male	4.8	1	2	Frequent	Splenectomized	88%	73%
BB-3	7	Male	5.9	2	3	Frequent	Splenectomized	92%	74%
BB-4	9	Female	6.9	1	1	Frequent	4	92%	72%

*Hemoglobin levels are obtained from the average hemoglobin level at a steady state or before receiving a blood transfusion. #The requirement for blood transfusion is defined by the frequency of transfusion, regular (every 2 weeks to 3 months), occasional (every 4 months to 1 year), and rare (more than 1 year). †Percentile of growth development is assessed based on weight and height measurements plotted on a Thai standard growth chart.

Supplemental Figure S1. Quantitative RT-PCR analysis of transcript levels of erythropoiesis modifying factors in day 8 (white bar) and day 10 (black bar) of culture as relative fold change with the mean and SD value from independent subjects of each thalassemia group compared to the normal group. N; normal, H; HbH disease, HCS; HbH-Constant Spring disease, BE; β^0-thalassemia/HbE disease, BB; homozygous β^0-thalassemia disease.

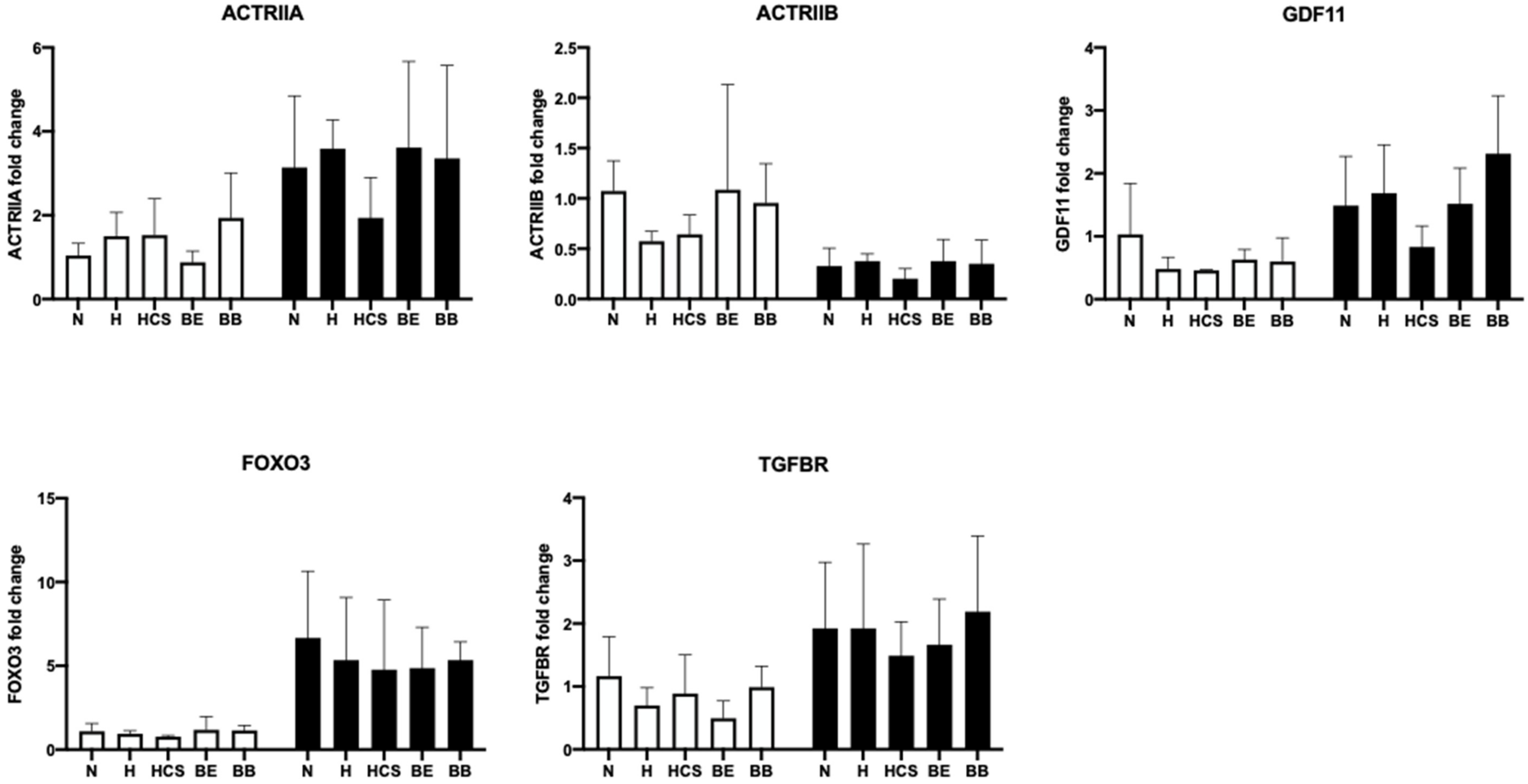

Supplement: Supplementary file 1 [file jcm-11-05356-s001.zip › jcm-1844438-supplementary.pdf]
